# Case report: A combination of nitroglycerin and adenosine proves effective in repairing a cerebral arteriovenous malformation

**DOI:** 10.3389/fneur.2023.1165155

**Published:** 2023-08-23

**Authors:** Virginia Rojas-Nieves, Cristian Rosa-Carrasquillo, Allan Reyes-Sullivan, Marie Román, Caleb E. Feliciano-Valls, Héctor M. Torres-Pérez, Pamela Fernández, María J. Crespo

**Affiliations:** ^1^Department of Anesthesiology, University of Puerto Rico School of Medicine, San Juan, Puerto Rico; ^2^Department of Neurosurgery, University of Puerto Rico School of Medicine, San Juan, Puerto Rico; ^3^Department of Physiology, University of Puerto Rico School of Medicine, San Juan, Puerto Rico

**Keywords:** arteriovenous malformations (AVM), adenosine, nitroglycerin, embolization, transvenous

## Abstract

Hemorrhage secondary to rupture of a brain arteriovenous malformations (AVM) is one of the initial manifestations, and the main cause of, morbidity and mortality in patients with this condition. Current treatment strategies include endovascular embolization with the goal of AVM obliteration and neurological preservation. In the transvenous endovascular embolization procedure, adenosine is the preferred agent to induce temporary hypotension and allow adequate AVM embolization. We describe the intraoperative management of an adenosine-resistant 38 year-old male who underwent a successful intracranial AVM embolization after concomitant administration of gradually increasing doses of nitroglycerin. This report suggests that nitroglycerin infusion can be combined with adenosine boluses to create a pronounced and dose-dependent hypotension in patients partially unresponsive to adenosine alone.

## Introduction

Intracranial arteriovenous malformations (AVMs) are defined as abnormal connections between the high-pressure arterial circulation and the low-pressure venous circulation (1). These connections increase venous pressure in AVMs, which contribute to their rupture and subsequent hemorrhage (2). AVMs may be discovered incidentally or as a result of various symptoms, including acute intracranial hemorrhaging, seizures, headaches, and focal neurological deficits. Although the mechanisms and contributing factors associated with AVM formation are still undefined, changes in genetic pathways and abnormal expression of inflammatory proteins have been linked to this pathology (3, 4). Endovascular techniques, invasive surgery, interventional radiosurgery, and conservative approaches have been used to lower the risk of AVM rupture and resultant hemorrhagic stroke (5). Endovascular embolization, which is a procedure that blocks blood flow through an AVM, has recently been demonstrated to successfully obliterate these malformations (6). This approach requires close access to the nidus, while maintaining low mean arterial pressure (MAP) to prevent backflow into the venous system and avoid occlusion of the venous drainage. The effectiveness of this procedure has become reliant on the use of vasodilators to induce time-sensitive periods of systemic hypotension, which allows the embolic agents to occlude the nidus without causing neurologic effects (6). Historically, the most common vasodilators are nitroglycerin, esmolol, nitroprusside and inhalational agents (6). All these agents cause hypotension individually, and their effects are not easily reversed should hemodynamic compromise occur. Since nitroprusside is associated with a risk for cyanide toxicity and esmolol infusions are expensive, nitroglycerin is a readily available option for intracranial aneurysm surgeries. By increasing nitric oxide release in vascular smooth muscle, nitroglycerin causes vasodilation that is easily titrated during an infusion. It is also known to cause headaches after prolonged infusion and refractory hypotension in cases of hypovolemia or cardiac dysfunction. For AVM endovascular embolization, however, literature on nitroglycerin use is insufficient. Adenosine has been proved to be a better alternative for controlled hypotension due to its replicability, consistency, and minimal side effects (7). This agent is a purine nucleoside that is rapidly cleared from blood with a short half-life of 0.6–20 s. A bolus of this drug causes flow arrest and decompresses the aneurysm sac, which in turn improves visualization and clipping without prolonged hypotension. By working directly at the AV node, adenosine in high doses may cause a high-grade conduction block, leading to arrythmias, hypotension and transient cardiac arrest. Thus, patients receiving adenosine should have cardiac arrest defibrillator pads placed prior to use in case a shockable rhythm develops. Tachyphylaxis induced by adenosine, however, has been described as a possible complication when administering increased doses for supraventricular tachycardia (7). In this report, we present a unique case of an adenosine-resistant patient treated with an effective combination of adenosine and increasing doses of nitroglycerine to achieve a target systemic MAP of 50 mmHg during an AVM embolization.

## Case presentation

**Figure 1:**
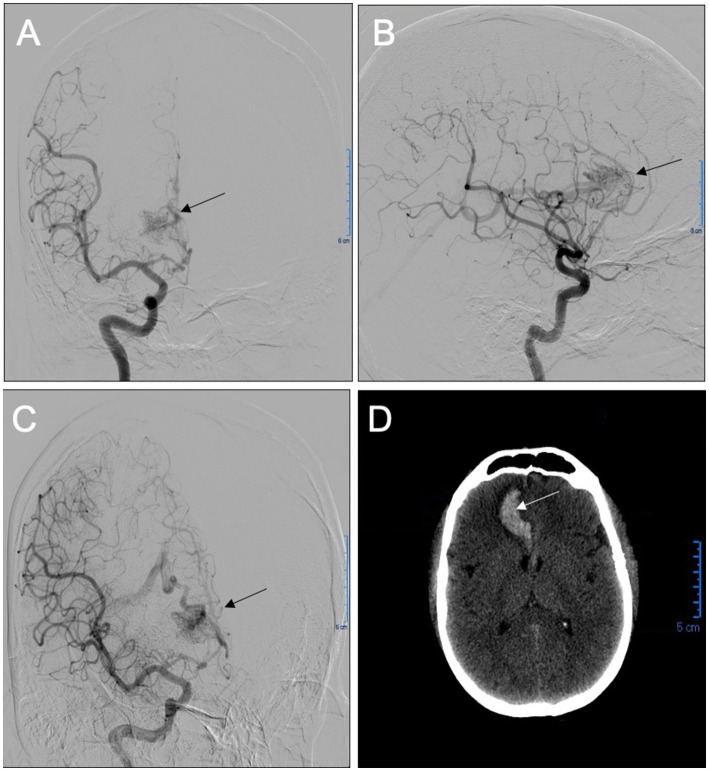
Anteroposterior **(A)**, lateral **(B)**, and oblique **(C)** projections of a preoperative digital subtraction angiography (DSA) from a right internal carotid artery injection demonstrated a diffuse right frontal arteriovenous malformation (AVM), with feeders from the right anterior cerebral artery and deep venous drainage. Preoperative head CT **(D)** shows an intraparenchymal hematoma centered in the right medial frontal lobe extending to the callosal sulcus.

**Figure 2:**
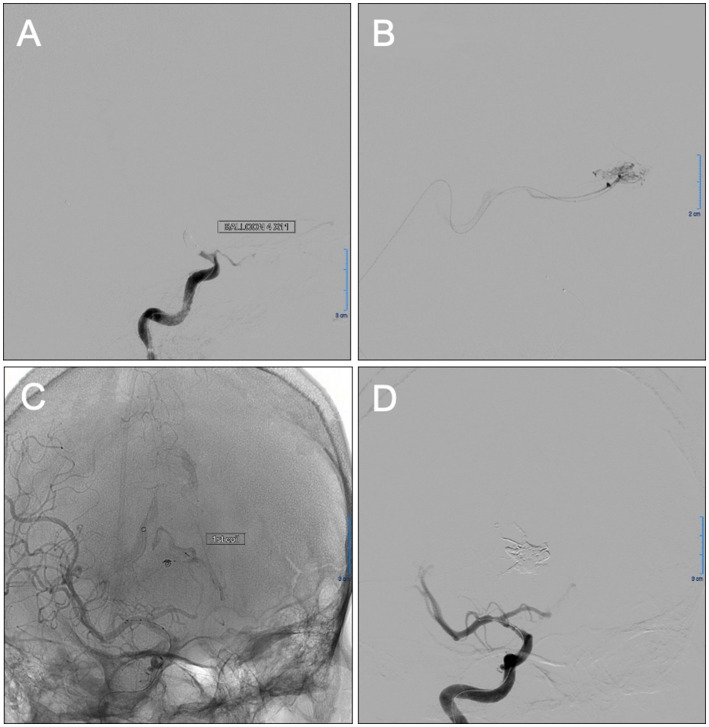
Intraoperative images showing planning and completion of the transvenous Onyx embolization of the diffuse right frontal arteriovenous malformation (AVM). Balloon test occlusion of the anterior cerebral artery A1 segment **(A)** was done during embolization for proximal vascular control of the AVM nidus flow. After superselective venous catheterization of the straight sinus, deep venous system, and right frontal AVM nidus, superselective runs **(B)** showed nidus configuration and no significant collateral reflux. Unsubstracted radiograph **(C)** shows proper placement of aneurysm coil proximal to the nidus performed under fluoroscopic guidance to reduce venous reflux of Onyx (pressure cooker technique). Oblique digital subtraction angiography (DSA) view of right internal carotid injection post-embolization **(D)** showed complete obliteration of the AVM nidus.

**Figure 3:**
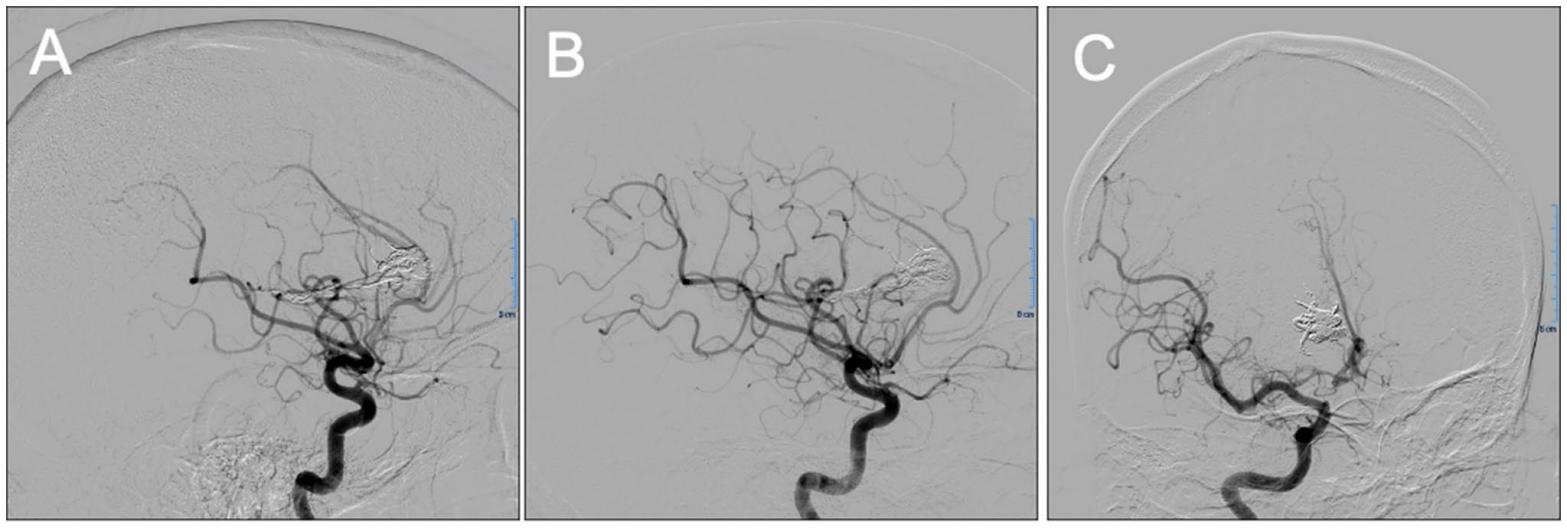
Lateral **(A,B)** and oblique **(C)** projections of control digital subtraction angiography (DSA) 10 months after embolization showing no evidence of arteriovenous malformation (AVM) recurrence.

The patient was a 38 year-old male with a past medical history of hypertension, which was adequately treated with oral nifedipine and enalapril. The patient’s chief complaint was a severe headache that was accompanied by one-sided weakness of his left side and slurred speech, dizziness, nausea, and episodes of emesis, which led him to seek medical attention. Upon evaluation, he had preserved cranial nerve function and mental status with a Glasgow Coma Scale (GCS) of 15/15. There was left-side hemiparesis graded 4 out of 5, as well as a positive Hoffman sign, left pronator drift, a negative Babinski sign, and hyperreflexia (+3) of the left side. A computed tomography (CT) without contrast of the head was performed that revealed a right frontal, flame-shaped, intracranial hemorrhage (ICH) involving the cingulate gyrus and corpus callosum. To identify the source of the bleeding, further imaging was performed with computed tomographic angiography (CTA) of the brain to evaluate any anomaly in the vascular system. Due to his young age and lack of risk factors for other more common etiologies of ICH, an AVM was suspected. The CTA confirmed the presence of a right medial frontal diffuse AVM (13.40 × 12.60 × 18.82 mm), with feeders from the right anterior cerebral artery, deep venous drainage, and an associated small perinidal aneurysm (Figure 1). Venous drainage involved the internal cerebral veins, vein of Galen, straight sinus to the torcula leading to the rest of the venous system. Using the Spetzler-Martin Grading system, which considers AVM size, eloquence, and location (8), the lesion was given a grade III. The supplementary Spetzler Martin Grade is 3 points given the age of patient (between 20 and 40), the presence of prior bleeding and its diffuse anatomic nature. The patient was immediately admitted into the neurosurgery intensive care unit (ICU) for closer monitoring and treatment. During his admission, his symptoms began to improve, but did not resolve completely. Since the ICH was estimated to be around 18 mL, and the patient was clinically stable, endovascular embolization was the preferred approach for this patient without prior hematoma evacuation. In preparation for the procedure, a systemic blood pressure threshold of 150/90 mmHg was maintained with enalapril (10 mg daily). In addition, any medications or activities that could secondarily increase intracranial pressure (ICP) were avoided. One week following the diagnosis and medical optimization, the patient was taken to the operating room for a cerebral angiogram and embolization of the AVM with ONYX embolic agent (Medtronic, Irvine, CA, United States), All ASA (American Society of Anesthesiologists) monitors were placed, including defibrillator patches. A 5 mg bolus of midazolam was administered (IV) as premedication. Smooth induction of anesthesia was achieved with 150 mg propofol, 100 μg fentanyl, and 100 mg succinylcholine. He was endotracheally intubated using direct laryngoscopy with MAC blade #4 and ETT size 7.5 mm. An additional peripheral IV line and a right arterial radial line were placed. Vascular access was gained through the left femoral artery and right femoral vein for local blood flow control with balloon insertion and embolisate injection, respectively. The arterial catheter setup included a Scepter C 4 mm X 11 mm (Microvention) compliant balloon microcatheter to assist with in-flow reduction to the AVM nidus. The venous catheter setup included a DMSO compatible (Headway Duo, Microvention) microcatheter. After micro catheterization proximal to the right frontal AVM’s nidus, the perinidal aneurysm was coiled under fluoroscopic guidance to reduce venous reflux of ONYX particles. The previously positioned balloon was inflated to obtain proximal arterial control and reduce forward blood flow. Following the method of Hashimoto and colleagues (9), a baseline nitroglycerin drip (5 mg/min) was initiated prior to the procedure in order to decrease MAP by 10% from baseline. Preceding AVM embolization, adenosine was used in boluses to induce short periods of target MAP (50 mm Hg that last at least 10 s). Specifically, an adenosine bolus of 12 mg (IV push) was administered, followed by two series of adenosine boluses. Each series consisted of an initial adenosine bolus (24 mg, IV push) followed by a second bolus (12 mg, IV push) after 30 s. Whereas in other patients the serial administration of adenosine induces transient hypotension, our patient did not achieve the expected hemodynamic response. In an effort to obtain the appropriate MAP reduction, the nitroglycerin drip was increased to 15 mg/min, yet the MAP remained above the target goal. Finally, the optimal MAP was achieved with an additional bolus of adenosine and an increase in nitroglycerin to 20 mg/min. AVM embolization was then performed by injecting a combination of ONYX 34 (0.6 mL) and ONYX 18 (1.0 mL). Complete obliteration of the arteriovenous defect was confirmed with a post-procedure angiogram (Figure 2). The only complication of the procedure was a transient reactive vasospasm at the carotid bifurcation during arterial access, which was treated with verapamil (3 aliquot of 5 mg/each). Paralysis was successfully reversed with sugammadex (200 mg), and morphine (4 mg) was then administered for postoperative pain. The patient was successfully extubated and transferred to the post anesthesia care unit (PACU) for observation and was later discharged to the ICU. A postoperative head CT showed moderate enlargement of intracerebral hemorrhage presumably due to adenosine resistance during procedure, which difficulted blood pressure control during the embolization requiring increased nitroglycerin dosing. Post-operative head CT after 24 h, showed hematoma size of around 30 cc as compared to 18 cc initially, however patient remained clinically stable with mild symptomatology. A second follow up head CT scan at 48 h showed stable ICH volume. Strict blood pressure control was employed at the ICU and he was subsequently discharged 5 days later to a rehabilitation facility. Ten months after the procedure, the patient presented to the neurosurgery clinics for a follow up angiogram, revealing no evidence of AVM recurrence (Figure 3). The patient’s outcome was favorable despite hemodynamic challenges, reporting resolved left hemiparesis and only mild balance deficit. Since the patient can carry out all daily without significant disability, a modified Rankin scale of 1 point was designated.

## Discussion

To our knowledge, this is the first description of the concomitant use of adenosine and nitroglycerin for the successful embolization of a brain AVM. Transvenous embolization has been associated with various complications, including AVM rupture and subsequent hemorrhage (10). Thickening and arterialization of the venous walls secondary to higher-than-normal intraluminal pressures, however, make this approach a safe alternative (11). Successful application of this method requires that the embolic agent overcomes the anterograde arterial pressure and move in a retrograde fashion from the venous system into the nidus. This procedure requires pharmacological intervention to achieve the necessary reduction in MAP.

Prior to the administration of adenosine, Hashimoto and colleagues (9) used a continuous infusion of nitroprusside to decrease baseline MAP by 10%. The baseline reduction of MAP is a useful strategy to reduce the potential for post-adenosine rebound hypertension (9). In the present report, however, nitroglycerin was selected as the vasodilator of choice because it has been associated with lower incidences of tachyphylaxis and rebound hypertension than nitroprusside (12, 13). Contrary to our expectations, repeated boluses of adenosine did not result in adequate hemodynamic control. The appropriate reduction in MAP was achieved, however, after increasing nitroglycerin infusion to a dose of 20 mg/min.

The use of adenosine alone to induce a transient arrest in the cardiac cycle during AV embolization has been described previously (6). Adenosine provides an advantage over other pharmacological agents due to its predictable duration, the extent of its effects, the reduced risk of complications, and its low side effects profile (9). Additionally, its short half-life allows for a profound and rapidly reversible transient hypotension, which increases the effectiveness of the embolic agent (6). The fact that adenosine was unable to effectively reduce MAP may indicate that this patient is adenosine resistant. It has been suggested that adenosine response and predictability are patient-dependent, and tachyphylaxis may develop after administration of repeated boluses of the drug (9, 14, 15).

After a complete AVM embolization by the transvenous route, the patient in this case showed no recurrence of his malformation in the 10 month, follow-up imaging. Such success rates following endovascular therapy alone are rare, even more so in challenging cases such as this one. The anesthetic management was of utmost importance during a procedure that relied heavily on hemodynamic control. Although the patient was only partially responsive to adenosine, nitroglycerin was able to act strategically as an adjunct by targeting a different component of the vascular response. In this way, greater control of the nidus pressures was attained, which in turn allowed for curative endovascular therapy. In conclusion, this report suggests that nitroglycerin infusion can be combined with adenosine boluses to create a pronounced and dose-dependent hypotension in patients partially unresponsive to adenosine alone.

One of the limitations of this pharmacological intervention, is that since this is a case report, the results were primarily based on a single adenosine resistant patient, who was only evaluated 10 months after the procedure. Further studies including more adenosine-resistant patients, and additional follow up interventions are needed to validate our conclusion. The findings of this study, however, provide a foundation to continue research on the concomitant use of adenosine and high dose nitroglycerine for successful embolization.

## Data availability statement

The raw data supporting the conclusions of this article will be made available by the authors, without undue reservation.

## Ethics statement

This study involving human participant was reviewed and approved by the Ethics Committee on Biomedical Research of the University of Puerto Rico- School of Medicine. Written informed consent to participate in this study was provided by the participant.

## Author contributions

VR-N worked in the conceptualization of the manuscript, writing the draft, and compiled the figures. CR-C and AR-S contributed with the writing and editing of the manuscript, and performed literature review. MR contributed with the writing of the draft and compilation of the figures. CF-V, HT-P, and PF revised manuscript and figures. MJC wrote the manuscript, revised the final version, and submitted the manuscript. All the authors read and approved the final version of the manuscript.

## Funding

This work was supported by the Anesthesiology Department of the University of Puerto Rico-School of Medicine.

## Conflict of interest

The authors declare that the research was conducted in the absence of any commercial or financial relationships that could be construed as a potential conflict of interest.

## Publisher’s note

All claims expressed in this article are solely those of the authors and do not necessarily represent those of their affiliated organizations, or those of the publisher, the editors and the reviewers. Any product that may be evaluated in this article, or claim that may be made by its manufacturer, is not guaranteed or endorsed by the publisher.
